# The Distribution of Sex Acts and Condom Use within Partnerships in a Rural Sub-Saharan African Population

**DOI:** 10.1371/journal.pone.0088378

**Published:** 2014-02-18

**Authors:** Jennifer Smith, Constance Nyamukapa, Simon Gregson, James Lewis, Sitholubuhle Magutshwa, Christina Schumacher, Phyllis Mushati, Tim Hallett, Geoff Garnett

**Affiliations:** 1 Department of Infectious Disease Epidemiology, Imperial College London, London, United Kingdom; 2 Biomedical Research and Training Institute, Harare, Zimbabwe; 3 MRC Tropical Epidemiology Group, London School of Hygiene and Tropical Medicine, London, United Kingdom; 4 Department of Pediatrics, Johns Hopkins School of Medicine, Baltimore, United States of America; 5 Global Development, Bill and Melinda Gates Foundation, Seattle, Washington, United States of America; University of Melbourne, Australia

## Abstract

**Introduction:**

In an HIV/AIDS epidemic driven primarily by heterosexual transmission, it is important to have an understanding of the human sexual behaviour patterns that influence transmission. We analysed the distribution and predictors of within-partnership sexual behaviour and condom use in rural Zimbabwe and generated parameters for use in future modelling analyses.

**Methods:**

A population-based cohort was recruited from a household census in 12 communities. A baseline survey was carried out in 1998–2000 with follow-up surveys after 3 and 5 years. Statistical distributions were fitted to reported within-partnership numbers of total, unprotected and protected sex acts in the past two weeks. Multilevel linear and logistic regression models were constructed to assess predictors of the frequency of unprotected sex and consistent condom use.

**Results:**

A normal distribution of ln(sex acts+1) provided the best fit for total and unprotected sex acts for men and women. A negative binomial distribution applied to the untransformed data provided the best fit for protected sex acts. Condom use within partnerships was predominantly bimodal with at least 88% reporting zero or 100% use. Both men and women reported fewer unprotected sex acts with non-regular compared to regular partners (men: 0.26 fewer every two weeks (95% confidence interval 0.18–0.34); women: 0.16 (0.07–0.23)). Never and previously married individuals reported fewer unprotected sex acts than currently married individuals (never married men: 0.64 (0.60–0.67); previously married men: 0.59 (0.50–0.67); never married women: 0.51 (0.45–0.57); previously married women: 0.42 (0.37–0.47)). These variables were also associated with more consistent condom use.

**Discussion:**

We generated parameters that will be useful for defining transmission models of HIV and other STIs, which rely on a valid representation of the underlying sexual network that determines spread of an infection. This will enable a better understanding of the spread of HIV and other STDs in this rural sub-Saharan population.

## Introduction

The HIV/AIDS epidemic in sub-Saharan Africa is primarily driven by heterosexual intercourse [1], [2] yet there is relatively little detailed information available on the characteristics and properties of human sexual behaviour in these populations. Modelling analyses are frequently used to understand the pattern of spread of the virus, plan prevention strategies and assess their utility and require a detailed understanding of these contact patterns in order to tailor analyses to a particular population, location or epidemic context, and to provide valid results [3]–[8].

The first studies that sought to quantify human sexual behaviour were carried out by Kinsey and colleagues in the 1940s and 1950s [9], [10]. Whilst their studies on sexual behaviour in the human male was well-received, the equivalent for women garnered widespread criticism [11] and was followed by a decades-long hiatus in research on human sexual behaviour. Interest was re-sparked in the 1980s by the early spread of HIV/AIDS, when it quickly became apparent that an understanding of the network of sexual partnership formation underlying the transmission of HIV and other sexually transmitted infections (STIs) was urgently required [12], [13].

In several developed countries, large-scale studies into sexual behaviour were undertaken in the early 1990s, including the UK [14], France [15] and the US [16]. Early studies in rural Africa, Asia and Central and South America took place contemporaneously with those in developed countries but generally recruited fewer participants [17]–[19]. Although there was large between-survey variation, these studies confirmed many hypothesised assumptions and provided new and important insights into human sexual behaviour. For example, in rural Tanzania, men reported a higher level of sexual activity and more lifetime partners than women, peak activity occurred in younger age groups, and the number of sex acts per partner per week declined with increasing number of partners in the past year [17]. Men also reported more non-marital sex than women, with up to 25% also reporting contacts with sex workers across 18 developing countries [18].

There are continuing concerns with reporting bias in self-reported sexual behaviour, which may distort estimates and comparison over space and time. In addition to inaccuracies with direct recall [20], perceived social norms may influence reported behaviour [21], and low participation rates and loss to follow-up may introduce participation bias, leading to validity issues with some datasets [22]–[24]. Although reported sexual behaviour does not balance between the sexes, it is believed that this is partially accounted for by women at the high extremes of the risk distribution [25], who may be omitted from general population surveys.

Condom use has been proven to reduce transmission of HIV and other STIs when used consistently and correctly, with an estimated 95% reduction in per-contact probability for HIV in heterosexual transmission [26], [27]. Over the past twenty years it has become the most widely used method of contraception in young single African women [28]. A number of studies have shown that condom use is more commonly reported in men than women [29]–[38], and that reported condom use is low within marriages [37]–[42]. All of these studies have documented reported condom use based on an instantaneous measure of condom use at last sex but, for the spread of STIs, patterns of intermittent use and temporal changes will also be important. Therefore, in this study, we focus on consistent condom use within partnerships, as measured by the proportion of total sex acts in the previous two weeks where a condom was used throughout.

In order for modelling analyses to provide valid results, they must be parameterised with data that are representative of local populations and reflect how behaviours are distributed rather than relying purely on simple averages. Whilst the risk of HIV transmission per sex act is considered low, estimated at a lower bound of 1 per 1000 sex acts [43], HIV risk within a partnership derives from the accumulation of sex acts over a period of time. Therefore, in order to fully characterise per-partnership risk, it is important to understand how and when unprotected sex acts accrue with individual partners. In this study we undertake an in-depth analysis of a decade of sexual behaviour data from the Manicaland HIV/STD Prevention Project, a well-documented cohort study in rural Zimbabwe [44]. We describe key parameters of the distribution of the number of sex acts within a partnership over a specified time interval and examine the distribution of within-partnership condom use and determinants of unprotected sex.

## Methods

### Ethics statement

Ethical approval for the study was granted by the Research Council of Zimbabwe (no. 02187), the Applied and Qualitative Research Ethics Committee in Oxford, UK (N97.039) and St. Mary's Local Research Ethics Committee, London (ICREC_9_3_13). Informed consent was obtained prior to participation in all surveys. Written informed consent was obtained from the next of kin, carers or guardians on the behalf of the participants aged 17 and under who were involved in the study.

### The Manicaland HIV/STD Prevention Project

The Manicaland HIV/STD Prevention Project is a population-based open cohort study conducted in 12 sites in the Manicaland province in eastern Zimbabwe. Details of the study procedures have previously been published elsewhere [36]. In brief, a baseline survey (termed round 1 (R1)) was carried out in 12 study sites in a phased manner between July 1998 and February 2000, with subsequent follow-up rounds conducted approximately 3 and 5 years later (termed R2 and R3). Study sites differ in terms of their socioeconomic location, comprising two small towns, four commercial forestry, tea and coffee estates, two roadside business centres (RBC), and four subsistence farming areas (SFA).

All members of households were initially enumerated in a census. Then, adults (men aged 17–54 years and women aged 15–44 years; expanded to include all men and women aged 15–54 years from R3) were invited to participate in the individual cohort study, in which all sexual behaviour data was collected. Only one person within each cohabiting marital union was eligible for the cohort study in R1 and R2. This was expanded to include both partners from R3; therefore, one individual from each marital couple was randomly excluded from the R3 data in this analysis in order to avoid any biases due to this change in protocol. Attempts were made to follow up all participants from the original cohort who were still resident in the study areas in subsequent rounds. Individuals reaching the upper age boundary during the study period were still included in subsequent rounds. Participation in the cohort required a face-to-face interview (FTFI) on a detailed questionnaire, which comprises an informal interview to establish rapport, with questions gradually progressing from straightforward to more private topics. Literacy among participants was very high (83% among men, 77% among women), and information was collected verbally from illiterate participants in either of the common languages Shona or Ndebele. Seventy-five per cent of literate respondents were randomly selected to complete the latter section on sexual behaviour by informal confidential voting interview (ICVI) in order to reduce social desirability bias [45]; respondents who were re-interviewed in subsequent rounds were reassigned to the same method. In ICVI, the respondent uses a secret voting strip to answer questions read by the interviewer, which is then deposited in a locked voting box. Individual participation rates were 79, 79 and 83% in the first, second and third surveys, respectively [46]. As previously reported, 56% of participants interviewed at R1, and not known to have died subsequently, were re-interviewed at [36], and 58% were re-interviewed from R2 to R3. The primary reason for drop-out between rounds was migration.

Within the sexual behaviour section of the individual questionnaire, data was collected on up to two of the participants' most recent sexual partners in R1 and R2, and three in R3, limited to those with whom the participant had had sexual relations in the past month. The time limit was removed for some later sites (R3 sites 7–12), so, for consistency with earlier rounds, data from these sites were not used in the current analysis. Individuals were asked to enumerate the number of times they had had sex with the specified partner in the previous two weeks, and the number of times they had used condoms throughout each coitus by secret voting, as well as other demographic and behavioural questions. Therefore partnership data from a single individual could be analysed both within a round and across different rounds (although the same partner cannot be identified across rounds); these two levels were amalgamated into one dataset of all partnerships. HIV prevalence was 16.4% among men and 22.0% among women in the general population over the time period of this study.

### Statistical methods

In this analysis we focus on three outcome measures of sexual behaviour: the total number of sex acts with a single partner in the past two weeks and the numbers of unprotected and protected sex acts in this period. For all analyses, ‘protected sex acts’ refers to those where condoms were used and the data are stratified by gender. Unprotected sex acts were calculated by subtracting the number of protected sex acts from the total number of sex acts.

Point estimates were calculated for all data. The non-parametric Mann-Whitney U test was used to test for differences in the distribution of unadjusted continuous variables and the χ^2^ test was used for categorical variables. Log-likelihood and Akaike information criterion (AIC) values were calculated to assess which distribution provided the best description for count data on the number of sex acts. We tested two distributions on the original data: the Poisson distribution for count data and the negative binomial to account for overdispersion; and we tested the normal distribution on a logarithmic transformation of the data offset by one, i.e. ln(sex acts+1).

Multilevel linear and logistic regression models were fitted for the reported number of unprotected sex acts in the previous two weeks and the proportion of total sex acts where a condom was reported to have been used throughout in the previous two weeks, respectively. We used an individual identifier as a random effect to control for participants who reported multiple partnerships within and between data collection rounds. All variables of interest were included in the multilevel models. For the reported number of unprotected sex acts in the previous two weeks these were: respondent age, age difference between partner and respondent, marital status, partnership type, reported partner number, data collection round, site type, education level, and HIV status. The total number of sex acts in the previous two weeks (protected and unprotected) was added to the potential predictors for consistent condom use model. All variables were categorical except age difference between partner and respondent and total number of sex acts. Interactions between marital status, partnership type, partner number and site type, and age of respondent and age difference between respondent and partner were also tested. Statistically significant (p<0.05 by ANOVA) interactions were retained in the final model.

Since the inclusion criterion for this section of the questionnaire is coitus within the last month, the denominator in all analyses is the sum of the individuals that report any sexual partners within the past month. All statistical analyses were performed using R version 2.12.1 [47]. All reported *P*-values are two-tailed and the confidence intervals (CI) at the 95% level.

## Results

### Data summary

In total, we report data on 11548 partnerships from 7836 individuals across the whole study population (Table 1). There are more partnerships than individuals because up to two recent sexual partners can be reported per person in R1 and R2, and three in R3. There are consistently more women than men throughout the study and women report a lower mean number of partners per person than men.

**Table 1:** Summary of data.

	Mean partners per individual (standard deviation, *total no. of partners*, no. of individuals)		
	Men	Women	Total
Round 1	1.13 (0.34, *2188*, 1929)	1.03 (0.16, *2516*, 2452)	1.07 (0.26, *4704*, 4381)
Round 2	1.12 (0.32, *1507*, 1348)	1.04 (0.19, *2147*, 2073)	1.07 (0.25, *3654*, 3421)
Round 3	1.12* (0.33, *1377*, 1196)	1.03* (0.16, *1813*, 1762)	1.07* (0.25, *3190*, 2958)
Total	NA (NA, *5072*, 3304)	NA (NA, *6476*, 4532)	NA (NA, 11548, 7836)

NOTE. NA, not applicable, as individuals may be resampled from round to round but partnerships are not; hence the number of individuals in each round also does not sum across all rounds.

* Data on up to two recent partners was collected in rounds 1 and 2, and up to three for round 3. For consistency, the third reported partner was omitted for the calculated mean partners per individual and standard deviation for round 3, where one existed (n = 34 for men and n = 2 for women).

### Distribution of sex acts in the past two weeks

**Figure 1:**
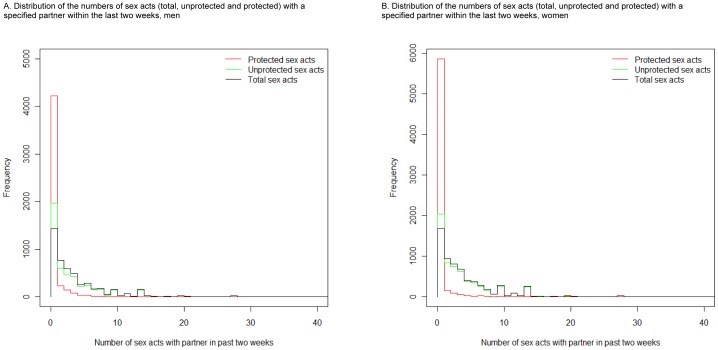
Overlaid histograms showing the distributions of the numbers of sex acts (total, unprotected and protected) with a specified partner within the last two weeks for (A) men and (B) women. Note that the line for total sex acts is lower than that for either unprotected or protected sex acts at zero because individuals reporting either zero unprotected or protected sex acts did not often report zero sex acts in total (due to the observed bimodal pattern of condom use described later).

Significant differences (p<0.001, Mann-Whitney U test) between men and women were found in the numbers of total, unprotected and protected sex acts in the past two weeks (Figures 1A and 1B). Among both men and women, no sex acts took place within the last two weeks for a substantial proportion of partnerships (13.4% and 14.6%, respectively). This suggests that the last sex act in these partnerships was 2–4 weeks previously. Few people report very high numbers of sex acts, yet there are still a large proportion reporting more than ten per week (12.1% and 13.6% for men and women, respectively). For both sexes, there is data heaping at 10, 14 and 28 sex acts, which represent 10 per fortnight and one or two per day, respectively.

The distribution of unprotected sex acts is similar to that for the total sex acts for both sexes, although this similarity is more pronounced in women. This is due to less condom use in women, with the proportion reporting no protected sex acts in the past two weeks higher for women (90.4%) than men (80.1%; Figure 1). For both sexes, few respondents report using condoms more than five times in the last two weeks (8.3% and 5.3% of those that report more than five sex acts for men and women, respectively).

For both men and women, there is a median of 3 total and 0 protected sex acts in the last two weeks. The median number of unprotected sex acts is 2 for men and 3 for women. These are lower than the corresponding mean values of 4.4 (95% confidence interval (CI) 4.2, 4.5), 3.8 (3.6, 3.9) and 0.61 (0.53, 0.69) sex acts in the previous two weeks for men and 4.5 (4.4, 4.6), 4.2 (4.0, 4.3) and 0.33 (0.29, 0.36) for women, for total, unprotected and protected, respectively (Table S1 in File S1).

**Figure 2:**
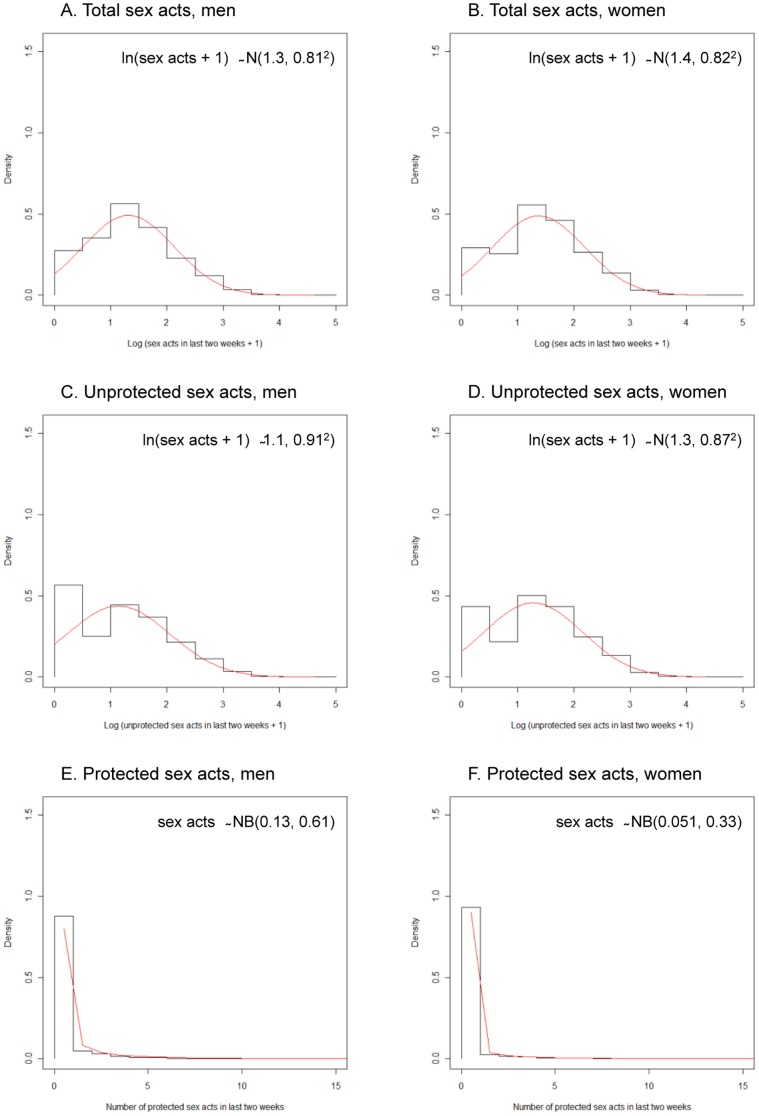
Observed and fitted distributions of the number of sex acts in the last two weeks. Panels A, C and E represent the numbers of sex acts (total, unprotected and protected) reported by men and B, D and F represent the same for women. A normal approximation of ln(sex acts+1) is used for A–D, and a negative binomial approximation of the untransformed data is used for E and F.

Different distributions were fitted to the data for each of the main sexual behaviour indicators examined (Table S2 in File S1). For total and unprotected sex acts, the normal approximation of ln(sex acts+1) was found to best represent the data; for protected sex acts, the best fit was given by the negative binomial distribution applied to the untransformed data. The fitted distributions are shown in Figure 2. As in Figure 1, the distributions for the numbers of total and unprotected sex acts in the previous two weeks are similar within and between the sexes, with the differences occurring at the low numbers.

### Total number of sex acts by gender and age

**Figure 3:**
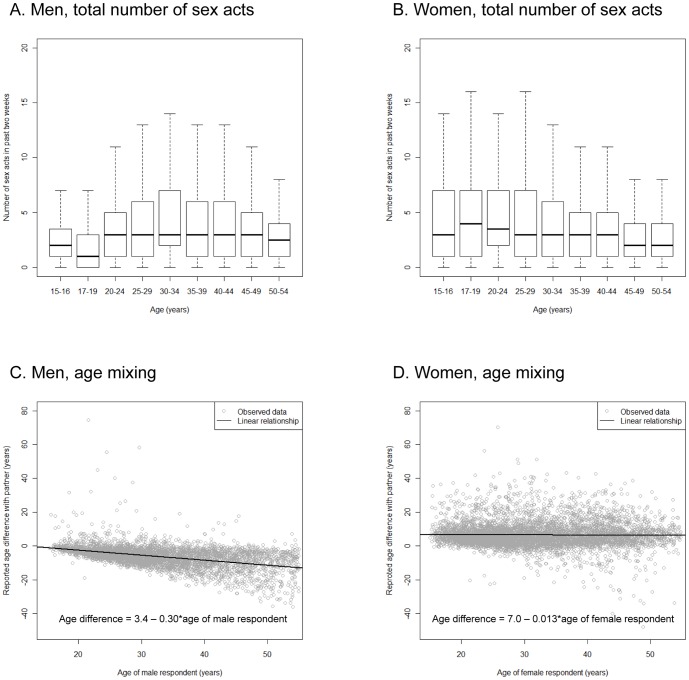
Partnership characteristics. A and B. Boxplots showing the total number of sex acts in the past two weeks by age for (A) men and (B) women. The heavy solid line marks the median and the box edges show the lower and upper quartiles. Whiskers show the minimum and maximum results which are no more than 1.5 multiplied by the interquartile range (IQR) from the box and outliers are not shown. C and D. Scatterplots showing the relationship between respondent age and age difference with partner reported by (C) men and (D) women. Solid lines mark the linear regression of respondent-partner age difference on respondent; these are in the form *y* = *mx*+*c*, where *x* is the respondent age and *y* is the respondent-partner age difference. For men (C), *m* = 0.30 (95% confidence interval [CI95] −0.31 to −0.29) and *c* = 3.4 (2.9–3.9). For women (D), *m* = −0.013 (−0.033–0.0065) and *c* = 7.0 (6.3–7.6).

The number of sex acts within a partnership varies by age (Figures 3A and B). For men, the median number of sex acts in the past two weeks decreases from 2 at age 15–16 to 1 at age 17–19 years then increases to a plateau of 3 from 20 years which continues until age 49 years. For women, teenagers aged 15–16 years report a median of 3 sex acts per fortnight within a partnership, increasing to a peak of 4 for 17–24 year-olds. This then declines with increasing age. The denominator in this analysis is sexually active individuals reporting sexual activity within a partnership in the past month, so it is important to note that this does not represent a peak of sexual activity for all 17–24 year-old women.

### Sexual mixing patterns by age

The observed pattern of sexual mixing is highly assortative with respect to age (Figures 3C and 3D, see Table S3 in File S1 for raw data). For men, variation in partner age increases with increasing age, so that older men tend to report a larger age difference between themselves and their partners. For women, the age difference between themselves and their partners remains constant with age, so that women of all ages report that their partners are approximately seven years older.

### Determinants of the frequency of unprotected sex within a partnership

Table 2 shows the association between the number of unprotected sex acts per partner per two weeks and a set of explanatory variables (column labelled ‘effect size’). In the baseline case, a 25–29 year-old HIV-negative male with secondary or higher education living on a forestry, tea or coffee estate in 1998–2000 engages in 2.8 (2.5, 3.1) unprotected sex acts per fortnight with his 25–29 year-old regular, marital partner of the same age whom he reports first in the questionnaire. This equates to 73 (65, 81) unprotected sex acts per year if a constant rate of sex is assumed. For an equivalent woman, these numbers are 3.2 (2.9, 3.5) unprotected sex acts per fortnight, or 83 (75, 91) per year. The effect sizes of most variables are of small magnitude.

**Table 2:** Multivariable linear mixed model showing the determinants of unprotected sex within a partnership.

	Men				Women			
	Effect size	95% CI	Individual *P*-value†	Overall *P*-value*	Effect size	95% CI	Individual *P*-value†	Overall *P*-value*
Intercept	2.8	2.5, 3.1	<0.001		3.2	2.9, 3.5	<0.001	
Age of respondent				<0.001				<0.001
15–16	−0.11	−0.35, 0.20	0.464		0.071	−0.10, 0.28	0.456	
17–19	−0.12	−0.22, 0.0072	0.049		0.11	0.0085, 0.22	0.037	
20–24	0.010	−0.068, 0.091	0.862		0.038	−0.031, 0.11	0.284	
25–29	.	.	.		.	.	.	
30–34	0.010	−0.065, 0.089	0.867		−0.059	−0.12, 0.012	0.101	
35–39	−0.11	−0.18, −0.027	0.009		−0.12	−0.19. −0.046	0.002	
40–44	−0.14	−0.22, −0.050	0.004		−0.13	−0.20, −0.054	<0.001	
45–49	−0.19	−0.27, −0.095	<0.001		−0.18	−0.25, −0.091	<0.001	
50–54	−0.23	−0.31, −0.12	<0.001		−0.22	−0.31, −0.10	<0.001	
Age difference between respondent and partner				0.197				0.619
	−0.0031	−0.0076, 0.0012	0.182		0.00050	−0.0025, 0.0036	0.779	
Marital status				<0.001				<0.001
Never married	−0.64	−0.67, −0.60	<0.001		−0.51	−0.57, −0.45	<0.001	
Currently married	.	.	.		.	.	.	
Previously married	−0.59	−0.67, −0.50	<0.001		−0.42	−0.47, −0.37	<0.001	
Partnership type				<0.001				<0.001
Regular	.	.	.		.	.	.	
Non-regular	−0.26	−0.34, −0.18	<0.001		−0.16	−0.23, −0.072	0.001	
Partner number				<0.001				<0.001
Partner 1	.	.	.		.	.	.	
Partner 2 (P2) or 3 (P3)	−0.30	−0.37, −0.22	<0.001		−0.27	−0.39, −0.13	<0.001	
Round				<0.001				<0.001
Round 1 (1998–2000)	.	.	.		.	.	.	
Round 2 (2001–2003)	0.13	0.072, 0.20	<0.001		0.068	0.015, 0.12	0.010	
Round 3 (2003–2005)	0.22	0.14, 0.29	<0.001		0.13	0.064, 0.20	<0.001	
Site type				<0.001				0.015
Estates	.	.	.		.	.	.	
Roadside trading centres	0.077	−0.022, 0.18	0.127		−0.085	−0.15, −0.015	0.016	
Subsistence farming areas	0.17	0.10, 0.25	<0.001		−0.084	−0.13, −0.035	0.001	
Towns	0.033	−0.038, 0.11	0.371		−0.046	−0.12, 0.030	0.244	
Education level				0.409				0.258
None or primary only	0.021	−0.034, 0.088	0.493		−0.032	−0.077, 0.017	0.181	
Secondary or higher	.	.	.		.	.	.	
HIV status				0.727				0.139
Negative	.	.	.		.	.	.	
Positive	−0.021	−0.073, 0.037	0.467		−0.039	−0.090, 0.013	0.147	
Marital status×partnership type				<0.001				NA
Never married×Non-regular	0.35	0.17, 0.56	<0.001		NA	-	-	
Previously married×Non-regular	0.49	0.10, 1.0	0.011		NA	-	-	
Marital status×Partner number				<0.001				NA
Never married×(P2 or P3)	0.38	0.15, 0.65	0.001		NA	-	-	
Previously married×(P2 or P3)	0.45	0.050, 1.2	0.090		NA	-	-	
Partnership type×partner number				<0.001				NA
Non-regular×(P2 or P3)	−0.20	−0.32, −0.034	0.014		NA	-	-	

Effect size refers to the effect of the variable in question on the number of unprotected sex acts in the previous two weeks with a single partner. The modal group (denoted by stop mark) was selected as the reference for all categorical variables. Effect sizes and 95% confidence intervals were estimated by Markov Chain Monte Carlo sampling.

NOTE. CI, confidence interval.

† *P*-values based on the t statistic with the upper bound for the degrees of freedom.

*P-values estimated by Likelihood Ratio Test.

All *P*-values are reported to three decimal places. All other results are reported to two significant figures.

The peak number of unprotected sex acts for men is spread over ages 20–34 years, which are not significantly different to the 25–29 year-old reference group. This is preceded by fewer reported sex acts for teenagers and followed by a progressive decline as the age of the respondent increases. For women, there is an overall decline with increasing age from age 17 onwards. Age difference between respondent and partner is not statistically significant for either partner and has a very small effect size. Never and previously married men and women reported significantly fewer unprotected sex acts per week compared to currently married individuals (for regular and most recently reported partners following inclusion of interaction terms). There are 0.26 per partner per fortnight (6.8 (4.6, 8.9) per partner per year) fewer unprotected sex acts with non-regular compared to regular partners for men (for currently married respondents with their most recent partner) and 0.16 (4.2 (1.9, 6.0)) fewer for women; this may reflect a shorter duration of the partnership, which were not measured here. The order in which the partnerships were reported also has a significant effect, with fewer sex acts reported in the second compared to the most recent partnership (for regular partners of currently married respondents). More unprotected sex acts were reported in R2 and R3 compared to R1 for both sexes. There were different associations between location and number of unprotected sex acts for men and women: men in SFAs reported 4.4 (2.6, 6.5) more unprotected sex acts per partner per year than those in estates; whereas women in RBCs and SFAs reported 2.2(0.39, 3.9) and 2.2 (0.91,3.4) fewer unprotected sex acts per partner per year, respectively, than those in estates. Education level and HIV status are non-significant for both men and women.

Interaction terms between marital status, partnership type and reported partner number were statistically significant for men only. Whilst currently married men report fewer sex acts with non-regular compared to regular partners, this pattern is reversed for never married and previously married individuals, who report 0.09 and 0.23 more sex acts in the past two weeks (this is the sum of the individual variable and interaction terms; 2.3 and 6.0 per year), respectively. Similarly, never and previously married men report 0.08 and 0.15 more sex acts in the past two weeks with less recent compared to most recent partners (2.1 and 3.9 more per year). The effect of partner recency is compounded for non-regular compared to regular partners, with 0.5 fewer sex acts reported in the previous two weeks for the less versus the most recent partner (13 fewer per year).

### Condom use within a partnership

**Figure 4:**
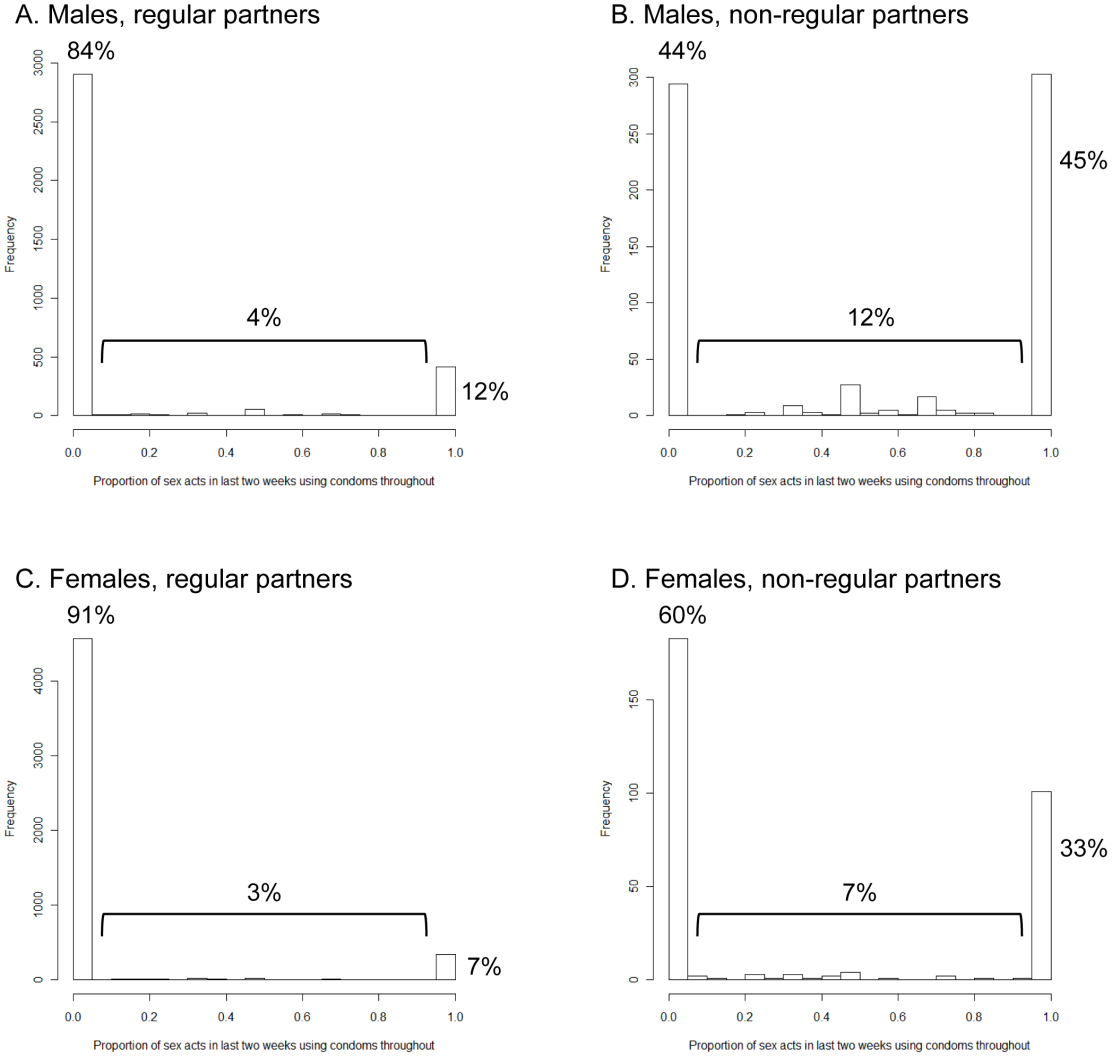
Histograms showing the proportion of sex acts in the last two weeks within a partnership in which a condom has been used throughout for men and women with regular and non-regular partners.

Condom use within a partnership is bimodal, with the vast majority of individuals using condoms either all the time or not at all within partnerships (Figure 4). A higher proportion of men than women report using condoms all the time (12 and 45% compared to 7 and 33% for regular and non-regular partners, respectively), but a stronger association is seen with type of partnership: a much higher proportion of both men and women always use condoms within non-regular compared to regular partnerships (p<0.0001 for both sexes by Chi-squared test). Irregular condom use is also reported more frequently with non-regular partners. However, even within non-regular partnerships, a majority of both men and women never use condoms.

### Determinants of consistent condom use within a partnership

Table 3 shows the association between consistent condom use (always using a condom compared to irregular or no use) and potential explanatory variables. Age of respondent is associated with consistent condom use for men and women, with adjusted analyses suggesting that men aged 17–24 and women aged 35–44 are more likely to always use condoms compared to the reference 25–29 year-old groups. Age difference between partner and respondent is statistically significant for women only, although the effect size is small (adjusted odds ratio (aOR) = 0.97 (0.95, 1.0)). The most powerful determinant of consistent condom use is marital status, with aORs of 16 (9.8, 26) and 19 (10, 35) for never married compared to currently married, and 28 (9.5, 81) and 16 (10, 25) for previously married individuals, for men and women respectively. Non-regular partnerships are significantly more associated with consistent condom use than regular partnerships, with aORs of 7.8 (5.0, 12) and 2.7 (1.6, 4.5) for men and women. As with unprotected sex acts, the order of reporting also has a significant effect, despite low numbers of people reporting two or three partners. Consistent condom use appears to have increased between R1 and R2 for women, and R1 and R3 for men overall. Site type has a statistically significant effect overall, although no one factor is significant for men. For women, consistent condom use is more common in towns compared to estates (aOR = 1.8 [1.1, 3.1]). Having no or primary level only education is associated with less consistent condom use compared to secondary or higher education for men only (aOR = 0.53 [0.37, 0.75]). For both sexes, HIV-positive status is associated with more consistent condom use (men: aOR = 1.5 [1.1, 2.1]; women: 1.6 [1.1, 2.4]).

**Table 3:** Generalised multivariable mixed model showing the determinants of consistent condom use within a partnership.

	Males				Females			
	Odds ratio	95% CI	Individual *P*-value†	Overall *P*-value*	Odds ratio	95% CI	Individual *P*-value†	Overall *P*-value*
Age of respondent				0.026				0.030
15–16	1.6	0.49, 5.4	0.424		1.4	0.33, 5.6	0.676	
17–19	2.4	1.4, 4.2	0.001		1.3	0.60, 2.7	0.512	
20–24	1.5	1.0, 2.3	0.031		0.93	0.52, 1.7	0.813	
25–29	1	.	.		1	.	.	
30–34	0.86	0.54, 1.4	0.529		1.3	0.73, 2.2	0.392	
35–39	1.1	0.62, 1.8	0.842		2.2	1.2, 4.0	0.013	
40–44	0.97	0.51, 1.9	0.937		2.0	1.0, 3.9	0.038	
45–49	0.60	0.26, 1.4	0.245		1.2	0.52, 2.9	0.647	
50–54	0.52	0.19, 1.4	0.203		0.50	0.086, 2.9	0.444	
Age difference between respondent and partner				0.103				0.004
	0.98	0.95, 1.0	0.112		0.97	0.95, 1.0	0.011	
Marital status				<0.001				<0.001
Never married	16	9.8, 26	<0.001		19	10, 35	<0.001	
Currently married	1	.	.		1	.	.	
Previously married	28	9.5, 81	<0.001		16	10, 25	<0.001	
Partnership type				<0.001				<0.001
Regular	1	.	.		1	.	.	
Non-regular	7.8	5.0, 12	<0.001		2.7	1.6, 4.5	<0.001	
Partner number				0.001				<0.001
Partner 1	1	.	.		1	.	.	
Partner 2 (P2) or 3 (P3)	1.8	1.3, 2.6	<0.001		4.0	1.9, 8.5	<0.001	
Round				0.088				0.001
Round 1 (1998–2000)	1	.	.		1	.	.	
Round 2 (2001–2003)	1.1	0.83, 1.5	0.450		1.6	1.1, 2.4	0.018	
Round 3 (2003–2005)	1.5	1.0, 2.1	0.033		2.1	1.3, 3.4	0.004	
Site type				0.004				0.002
Estates	1	.	.		1	.	.	
Roadside trading centres	1.7	0.84, 3.6	0.137		1.0	0.57, 1.9	0.894	
Subsistence farming areas	0.60	0.34, 1.1	0.077		0.67	0.42, 1.1	0.103	
Towns	0.69	0.40, 1.2	0.198		1.8	1.1, 3.1	0.032	
Education level				<0.001				0.838
None or primary only	0.53	0.37, 0.75	<0.001		0.97	0.65, 1.4	0.858	
Secondary or higher	1	.	.		1	.	.	
HIV status				0.016				0.008
Negative	1	.	.		1	.	.	
Positive	1.5	1.1, 2.1	0.021		1.6	1.1, 2.4	0.016	
Reported total number of sex acts in previous two weeks				<0.001				<0.001
	0.86	0.82, 0.90	<0.001		0.91	0.86, 0.95	<0.001	
Marital status×partnership type				<0.001				NA
Never married×Non-regular	0.12	0.066, 0.21	<0.001		NA	-	-	
Previously married×Non-regular	0.16	0.046, 0.54	0.003		NA	-	-	
Marital status×Site type				0.031				NA
Never married×Roadside trading centres	1.3	0.48, 3.3	0.637		NA	-	-	
Previously married×Roadside trading centres	0.39	0.070, 2.2	0.279		NA	-	-	
Never married×Subsistence farming areas	2.0	0.94, 4.1	0.071		NA	-	-	
Previously married×Subsistence farming areas	0.21	0.038, 1.2	0.081		NA	-	-	
Never married×Towns	3.2	1.4, 7.3	0.007		NA	-	-	
Previously married×Towns	0.86	0.17, 4.4	0.858		NA	-	-	

Odds ratios refer to the odds of always using condoms compared to sometimes or never using condoms during sex acts for the previous two weeks with a single partner, estimated by adaptive Gauss-Hermite approximation using ten integration points. The modal group (denoted by a stop mark) was selected as the reference for all categorical variables.

NOTE. CI, confidence interval.

† *P*-values are estimated by Wald test.

*P-values estimated by Likelihood Ratio Test.

All *P*-values are reported to three decimal places. All other results are reported to two significant figures.

Interaction terms between marital status and partnership type, and marital status and site type were statistically significant for men only. The influence of partnership type on consistent condom use is attenuated for never and previously married compared to currently married men, with overall aORs of 1.9 and 4.5 for non-regular versus regular partners, respectively (calculated by multiplying the aORs for the individual variable and the interaction terms). Only one factor is statistically significant in the marital status and site type interaction, indicating that the impact of never having married on consistent condom use is augmented for men in towns compared to estates.

## Discussion

In this analysis, we have described the distribution of sex acts (total, unprotected and protected) with a single partner within a specified time interval. This approach differs from most previous estimates of condom use, which look at instantaneous condom use at the single most recent sex act. Although condom use at most recent coitus has been reported to be highly correlated with consistent use [48], [49], it is useful to have an idea of the underlying distribution of sex acts between pairs of partners in order to accurately parameterise mathematical models of STI/HIV transmission based on sexual networks. We demonstrate that the number of total and unprotected sex acts follow a similar distribution, because consistent condom use has been low overall in this rural sub-Saharan African population.

Although the number of sex acts in two weeks may be hypothesised to follow a Poisson or negative binomial distribution, log-likelihood analysis shows that the normal approximation of a logarithmic transformation of the data provides a better fit for counts of total and unprotected sex acts. Protected sex acts, where a condom was used, are more accurately modelled with the negative binomial distribution. This allows for a greater influence of over-dispersion, perhaps due to the highly zero-inflated nature of the data. Men report more partners overall (Table 1) but fewer sex acts per partner per fortnight (Table 2). This may reflect a higher number of non-regular partners [18]. The mean total number of sex acts of 4.4 and 4.5 per two weeks reported here is slightly higher than the 7.9 per month among married women who have had sex in the past month reported in the 1994 Demographic Health Survey (DHS) for Zimbabwe [50]. The observed pattern of age mixing, which does not change through time, is consistent with previous analyses [51].

The major predictor for the number of unprotected sex acts was marital status, as noted in previous studies [37]–[42]. The increasing number of unprotected sex acts reported over time may not reflect a true behavioural change but a cohort effect, where participants became more comfortable with answering personal questions after the initial baseline survey (R1) [45]. The elevated number of unprotected sex acts for men in SFAs compared to estates may be due to increased cohabitation with a long-term partner, and therefore more regular contact. The reversal of this trend among women may reflect the absence of partners due to migrant work. In RBCs, a higher proportion of migrant workers, divorcees and widows may reduce their regular sexual contacts resulting in the fewer unprotected sex acts reported by women [52].

The majority of both sexes never use condoms, particularly with regular partners, although consistent use is more reported more commonly by men than women. Within-partnership condom use has a bimodal distribution, with the majority of people using condoms all the time or never. This is analogous to a ‘take’ rather than a ‘degree’ type vaccine, which confers full protection to a proportion of recipients. This is an important distinction from a mathematical modelling perspective as it results in a proportion of the population being fully protected, hence removed from the pool of individuals who are susceptible to infection. This may affect the distribution of those infected and thus the effectiveness of any interventions [53] and would thus be an important addition to a partnership-based model. However it is possible that the observed bimodal distribution was augmented by social desirability bias [22], with some individuals reporting condom use in all sexual encounters when in reality condoms were used in only a majority.

It has been documented previously that condom use in similar populations is associated with gender, age and partnership type [29]–[38]. Here we report that the most important determinants of condom use are marital status, partnership type and the reported partner order for both women and men. The latter may be a methodological distinction, where participants list their spouse or regular partner before any extra-marital relationships, or it may reflect a higher coital frequency with regular compared to non-regular partners. The pattern of age-related consistent condom use in women is non-intuitive. We would expect to find a decrease in condom use with increasing age but find almost the opposite. This may reflect some social desirability bias, which may differ by age, with a disproportionately low number of older, monogamous women, who do not commonly use condoms, likely to report details of their sexual behaviour, or because there are disproportionately more widows and sex workers among older women who are more likely to engage in high-risk partnerships where condom use is more frequent [54]. These sexual behaviour data are collected over an eight year period and amalgamated, hence the collection round was explicitly included as a possible explanatory variable in the multivariate analysis. We observe an increase in consistent condom use over time, occurring earlier for women than men. This is consistent with other reports, which have indicated that condom use has increased in sub-Saharan Africa, especially in among younger people [28], [55]. The association of primary education level with less consistent condom use for men reflects the higher HIV prevalence in this population [56]. These results suggest younger women with an older partner or older men who are less educated are the least likely to use condoms consistently, potentially due to omission from or resistance to intervention efforts.

These data provide a valuable insight into the pattern of sexual behaviour within a relationship in a sub-Saharan African population. However, an equally important determinant of how a sexually transmitted disease spreads through a population is the nature of the sexual network, for example rate of partner turnover, the distribution of the number of partners, and prevalence of concurrent partnerships; these data will also be important for model parameterisation. We have generated parameters that will be useful for defining transmission models of HIV and other STIs, which rely on a valid representation of sexual behaviour that determines spread of an infection. This will enable a better understanding of the spread of HIV and other STDs in this rural sub-Saharan population.

## Supporting Information

File S1 **Combined supporting information.** Table S1. Summary statistics. Table S2. Fitting distributions to data. Table S3. **A.** Female partner age reported by male respondent. **B.** Male partner age reported by female respondent.
(DOCX)
