# Supplementary material for: The Distribution of Sex Acts and Condom Use within Partnerships in a Rural Sub-Saharan African Population
Source: PLoS One. 2014 Feb 18;9(2):e88378. doi: 10.1371/journal.pone.0088378 (PMC3928170; doi:10.1371/journal.pone.0088378)
Supplement: File S1 — Combined supporting information. Table S1. Summary statistics. Table S2. Fitting distributions to data. Table S3. A. Female partner age reported by male respondent. B. Male partner age reported by female respondent. (DOCX) [file pone.0088378.s001.docx]

**Combined Supporting Information**

**Table S1. Summary statistics**

|  | Total sex acts | |  | Unprotected sex acts | |  | Protected sex acts | |
| --- | --- | --- | --- | --- | --- | --- | --- | --- |
|  | Males | Females |  | Males | Females |  | Males | Females |
| Number of observations | 4818 | 6302 |  | 4807 | 6286 |  | 4813 | 6297 |
| Mean | 4.4 | 4.5 |  | 3.8 | 4.2 |  | 0.61 | 0.33 |
| CI95 | 4.2, 4.5 | 4.4, 4.6 |  | 3.6, 3.9 | 4.0, 4.3 |  | 0.53, 0.69 | 0.29, 0.36 |
| Median | 3 | 3 |  | 2 | 3 |  | 0 | 0 |
| SD | 5.6 | 4.9 |  | 5.3 | 4.9 |  | 2.7 | 1.5 |
| Kurtosis | 67 | 21 |  | 42 | 21 |  | 783 | 119 |
| Skewness | 5.4 | 2.9 |  | 4.1 | 2.9 |  | 23 | 8.8 |

NOTE. CI95, 95% confidence interval, SD, standard deviation.

All statistics refer to the number of relevant sex acts within a partnership within the previous two weeks. Mean unprotected and protected sex acts should sum to the mean total but there are differing levels of missing data.

**Table S2. Fitting distributions to data**

|  | Distribution *(data)* |  | Total sex acts | | |  | Unprotected sex acts | | |  | Protected sex acts | | |
| --- | --- | --- | --- | --- | --- | --- | --- | --- | --- | --- | --- | --- | --- |
|  |  |  | logLik | AIC | Parameters |  | logLik | AIC | Parameters |  | logLik | AIC | Parameters |
| Males | |  |  |  |  |  |  |  |  |  |  |  |  |
|  | Negative binomial  *(sex acts)* |  | -12000 | 25000 | size = 1.2,  mu = 4.4 |  | -12000 | 23000 | size = 0.69,  mu = 3.8 |  | -4200 | 8400 | size = 0.13,  mu = 0.61 |
|  | Poisson  *(sex acts)* |  | -18000 | 36000 | lambda = 4.4 |  | -18000 | 37000 | lambda = 3.8 |  | -7500 | 15000 | lambda = 0.61 |
|  | Normal  *(ln (sex acts + 1))* |  | -6100 | 12000 | mean = 1.3,  sd = 0.81 |  | -6400 | 13000 | mean = 1.1,  sd = 0.91 |  | -4400 | 8900 | mean = 0.29,  sd = 0.58 |
|  |  |  |  |  |  |  |  |  |  |  |  |  |  |
| Females | |  |  |  |  |  |  |  |  |  |  |  |  |
|  | Negative binomial  *(sex acts)* |  | -16000 | 33000 | size = 1.2,  mu = 4.5 |  | -16000 | 32000 | size = 0.96,  mu = 4.2 |  | -3300 | 6500 | size = 0.051,  mu = 0.33 |
|  | Poisson  *(sex acts)* |  | -22000 | 45000 | lambda = 4.5 |  | -23000 | 46000 | lambda = 4.2 |  | -6400 | 13000 | lambda = 0.33 |
|  | Normal  *(ln (sex acts + 1))* |  | -7800 | 16000 | mean = 1.4,  sd = 0.82 |  | -8100 | 16000 | mean = 1.3,  sd = 0.87 |  | -4100 | 8200 | mean = 0.15,  sd = 0.46 |

NOTE. logLik, log-likelihood, AIC, Akaike Information Criterion. All results are accurate to two significant figures.

**Table S3**

1. **Female partner age reported by male respondent**

|  | Age group of female partner (years) | | | | |  |  |  |  |  |  |
| --- | --- | --- | --- | --- | --- | --- | --- | --- | --- | --- | --- |
| Age group of male respondent (years) | <15 | 15-16 | 17-19 | 20-24 | 25-29 | 30-34 | 35-39 | 40-44 | 45-49 | 50-54 | 55+ |
| 15-16 | 5 | 15 | 7 | 0 | 2 | 2 | 0 | 0 | 0 | 0 | 0 |
| 17-19 | 15 | 136 | 148 | 25 | 2 | 2 | 2 | 0 | 0 | 1 | 0 |
| 20-24 | 10 | 103 | 472 | 344 | 42 | 8 | 3 | 1 | 0 | 1 | 3 |
| 25-29 | 1 | 26 | 207 | 563 | 241 | 18 | 9 | 0 | 1 | 3 | 3 |
| 30-34 | 1 | 5 | 57 | 273 | 324 | 127 | 25 | 3 | 1 | 0 | 0 |
| 35-39 | 2 | 3 | 12 | 94 | 188 | 186 | 97 | 7 | 2 | 0 | 1 |
| 40-44 | 0 | 1 | 6 | 28 | 74 | 102 | 164 | 61 | 3 | 2 | 2 |
| 45-49 | 0 | 1 | 4 | 20 | 35 | 49 | 86 | 98 | 46 | 1 | 2 |
| 50+ | 0 | 1 | 3 | 10 | 17 | 19 | 35 | 53 | 82 | 19 | 3 |

1. **Male partner age reported by female respondent**

|  | Age group of male partner (years) | | | | |  |  |  |  |  |  |
| --- | --- | --- | --- | --- | --- | --- | --- | --- | --- | --- | --- |
| Age group of female respondent (years) | <15 | 15-16 | 17-19 | 20-24 | 25-29 | 30-34 | 35-39 | 40-44 | 45-49 | 50-54 | 55+ |
| 15-16 | 0 | 4 | 15 | 46 | 17 | 3 | 5 | 2 | 1 | 2 | 0 |
| 17-19 | 0 | 0 | 14 | 188 | 170 | 39 | 13 | 9 | 6 | 2 | 1 |
| 20-24 | 3 | 0 | 3 | 232 | 552 | 279 | 94 | 17 | 25 | 8 | 9 |
| 25-29 | 2 | 0 | 0 | 26 | 236 | 514 | 290 | 71 | 43 | 19 | 31 |
| 30-34 | 1 | 1 | 3 | 12 | 38 | 194 | 362 | 186 | 89 | 39 | 43 |
| 35-39 | 0 | 1 | 2 | 3 | 13 | 31 | 137 | 300 | 151 | 73 | 80 |
| 40-44 | 1 | 0 | 3 | 3 | 4 | 15 | 15 | 116 | 221 | 162 | 162 |
| 45-49 | 3 | 2 | 3 | 0 | 2 | 2 | 11 | 9 | 86 | 159 | 186 |
| 50+ | 0 | 0 | 0 | 2 | 0 | 0 | 2 | 2 | 3 | 27 | 149 |
